# Selection Strategy of Jaw Tracking in VMAT Planning for Lung SBRT

**DOI:** 10.3389/fonc.2022.820632

**Published:** 2022-02-08

**Authors:** Wuji Sun, Yinghua Shi, Yu Li, Chao Ge, Xu Yang, Wenming Xia, Kunzhi Chen, Libo Wang, Lihua Dong, Huidong Wang

**Affiliations:** ^1^ Department of Radiation Oncology and Therapy, The First Hospital of Jilin University, Changchun, China; ^2^ Jilin Provincial Key Laboratory of Radiation Oncology and Therapy, Department of Radiation Oncology and Therapy, The First Hospital of Jilin University, Changchun, China; ^3^ National Health Commission (NHC) Key Laboratory of Radiobiology, School of Public Health, Jilin University, Changchun, China

**Keywords:** stereotactic body radiation therapy, jaw tracking technique, volumetric modulated arc therapy, planning target volume, small field

## Abstract

**Purpose:**

This study aimed to investigate the dosimetric effect and delivery reliability of jaw tracking (JT) with increasing planning target volume (PTV) for lung stereotactic body radiation therapy (SBRT) plans. A threshold of PTV was proposed as a selection criterion between JT and fixed-jaw (FJ) techniques.

**Methods:**

A total of 28 patients with early-stage non-small-cell lung cancer were retrospectively included. The PTVs ranged from 4.88 cc to 68.74 cc, prescribed with 48 Gy in four fractions. Three-partial-arc volumetric modulated arc therapy (VMAT) plans with FJ and with JT were created for each patient with the same optimization objectives. These two sets of plans were compared using metrics, including conformity index (CI), V_50%_, R_50%_, D_2cm_, dose–volume parameters of organs at risk, and monitor units (MUs). The ratio of small subfields (<3 cm in either dimension), %SS, was acquired as a surrogate for the small-field uncertainty. Statistical analyses were performed to evaluate the correlation between the differences in these parameters and the PTV.

**Results:**

The V_50%_, R_50%_, D_2cm_, and V_20Gy_, D_1,500cc_, and D_1,000cc_ of the lung showed a statistically significant improvement in JT plans as opposed to FJ plans, while the number of MU in JT plans was higher by an average of 1.9%. Between FJ and JT plans, the PTV was strongly correlated with the differences in V_50%_, moderately correlated with those in V_20Gy_ of the lung, and weakly correlated with those in D_2cm_ and D_1,500cc_ of the lung. By using JT, %SS was found to be negatively correlated with the PTV, and the PTV should be at least approximately 12.5 cc for an expected %SS <50%, which was 15 cc for a %SS <20% and 20 cc for a %SS <5%.

**Conclusions:**

Considering the dosimetric differences and small-field uncertainties, JT could be selected using a PTV threshold, such as 12.5, 15, or 20 cc, on the basis of the demand of delivery reliability for lung SBRT.

## Introduction

Stereotactic body radiation therapy (SBRT) has been widely used as an alternative modality to surgery for the treatment of early-stage non-small-cell lung cancer (NSCLC) in recent years (1–3). SBRT features a high dose delivered in few fractions, and a rapid dose fall-off away from the target and optimal target dose conformity are crucial to minimize the normal tissue toxicity (4–8). SBRT demands a high level of delivery accuracy during the treatment, which can be affected by multiple factors, such as beam modeling and machine commissioning (8, 9).

With the extensive implementation of SBRT, interest in the use of small photon fields has been rapidly growing (9–11). However, due to lack of lateral charged-particle equilibrium, collimator effects, and limited choices of radiation detectors, small-field dosimetry has always been challenging, and large uncertainties are generally expected in small-field measurements (12–14). Therefore, small-field dosimetry is one of the main factors related to the delivery accuracy of SBRT. In the commissioning of treatment planning systems (TPSs), such as the Eclipse TPS, the beam data for small fields, i.e., below 3 × 3 cm^2^, are mostly not included in the beam modeling, except for the carefully measured output factors, which could be used for jaw-defined fields (15).

Volumetric modulated arc therapy (VMAT), as an advanced radiation delivery technique involving simultaneous modulation of the multi-leaf collimator (MLC), dose rate, and gantry speed, could deliver highly conformed doses to the target while minimizing the dose to normal tissues, hence commonly used in SBRT (16, 17). Over the past few years, the jaw tracking (JT) technique has been broadly used for VMAT planning to reduce inter-leaf leakage and intra-leaf transmission through MLC (18, 19). Several studies demonstrated that using JT rather than fixed-jaw (FJ) technique could lead to improved organ-at-risk (OAR) sparing and superior dose fall-off away from the target (19–22).

The JT technique features continuous adjustment of jaw positions to fit the MLC apertures throughout the treatment process. In small-field planning, especially for SBRT, the use of JT could result in a large number of subfields below 3 × 3 cm^2^, which may cause large discrepancies between calculated and delivered doses (23–25). In the current clinical practice, the FJ technique is generally recommended for small-field planning to achieve minimal calculation uncertainties (26, 27). However, the decision-making between JT and FJ is usually based on users’ experiences, and a clear consensus is yet to be established. Besides, the correlation between the PTV and benefits from using JT is worth investigating.

This study aimed to investigate the dosimetric advantages and the ratio of small subfields while implementing JT in VMAT planning for lung SBRT with a wide range of tumor sizes and discuss the feasibility of using PTV as a selection criterion between JT and FJ techniques.

## Materials and Methods

### Patient Immobilization and Simulation

A total of 28 patients with early-stage peripheral NSCLC treated at the First Hospital of Jilin University between 2017 and 2021 were retrospectively included. The distances between the PTV and the chest wall were <0.5 cm for 12 patients, 0.5−1.0 cm for 6 patients, 1.0−1.5 cm for 6 patients, and >1.5 cm for 4 patients. Patients were immobilized with custom-made negative-pressure vacuum cushions in supine position. Four-dimensional computed tomography (4D-CT) scan was acquired on a Philips Brilliance Big Bore CT scanner with a bellows system (Philips Healthcare, Cleveland, OH, USA). The 4D-CT datasets were reconstructed as 10 respiratory phases with 1.5-mm slice thickness and then transferred to the Varian Eclipse 15.6 TPS.

### Target Definition

Maximum intensity projection (MIP) and average intensity projection (AIP) images were reconstructed in Eclipse. The internal target volume (ITV) was contoured on the MIP and reviewed on each phase of the 4D-CT dataset. The PTV was defined by adding an isotropic 5-mm margin to the ITV. The OARs were contoured on the AIP, including the lung, spinal cord, esophagus, and trachea.

### Treatment Planning

In accordance with the Radiation Therapy Oncology Group (RTOG) 0915 criteria (6, 7), a total dose of 48 Gy in four fractions was prescribed for all patients. Two VMAT-SBRT plans with three partial arcs (210°) were generated on the AIP for each patient, that is, one with fixed jaws (FJ plan) and the other with jaw tracking (JT plan). The collimator angles were set to 10°, 350°, and 80° for the three arcs of all plans. All plans were optimized using the same objectives and constraints. An upper monitor unit (MU) constraint of 3,500 MU and a moderate Aperture Shape Controller (ASC) setting were used to limit the plan complexity. It should be noted that the jaw-defined fields for FJ plans were all above 3 × 3 cm^2^. Plan optimization was performed in Eclipse version 15.6 TPS for a Varian VitalBeam linear accelerator equipped with a Millennium 120 MLC (Varian Medical Systems, Palo Alto, CA) using 6 MV FFF beam. The MLC transmission factor and the dosimetric leaf gap for 6 MV FFF were 1.17% and 0.14 cm, respectively. The maximum dose rate was 1,400 MU/min. The AcurosXB algorithm was used to calculate the dose distributions with a grid spacing of 1.25 mm. All plans were normalized to achieve the same target coverage, i.e., 95% of the PTV received 100% prescription dose.

### Plan Evaluation

The dose–volume parameters of FJ and JT plans were compared, including the following metrics per RTOG 0915 recommendations (6, 7): conformity index (CI), defined as the ratio of prescription isodose volume (V_100%_) to the PTV; ratio of 50% prescription isodose volume to PTV, R_50%_; maximum dose 2 cm away from the PTV (D_2cm_, in percentage of the prescription dose); percentage of lung receiving at least 20 Gy (V_20Gy_); doses to 1,500 cc (D_1,500cc_) and 1,000 cc (D_1,000cc_) of normal lung; and near-maximum doses to the spinal cord (D_0.35cc_), esophagus (D_5cc_), and trachea (D_4cc_). Besides, considering the uncertainty in the small-field dosimetry, the percentage of small subfields (<3 cm in either dimension), %SS, was calculated for each JT plan. The correlation between %SS and the PTV was analyzed.

### Data Analysis and Statistics

Wilcoxon signed-rank tests were used for the comparison between FJ and JT plans. Correlations between the PTV and differences in plan metrics between FJ and JT plans and those between the PTV and %SS were examined using Spearman’s rank correlation coefficient (*r*_s_). |*r*_s_| ≥ 0.7 was considered a strong correlation, 0.7 > |*r*_s_| ≥ 0.5 was considered a moderate correlation, 0.5 > |*r*_s_| ≥ 0.3 was considered a weak correlation, and |*r*_s_| < 0.3 was considered no correlation. A two-tailed *p* < 0.05 was considered statistically significant. All statistical analyses were performed using IBM SPSS Statistics 26.0 software (IBM Corporation, Armonk, NY, USA).

## Results

**Figure 1:**
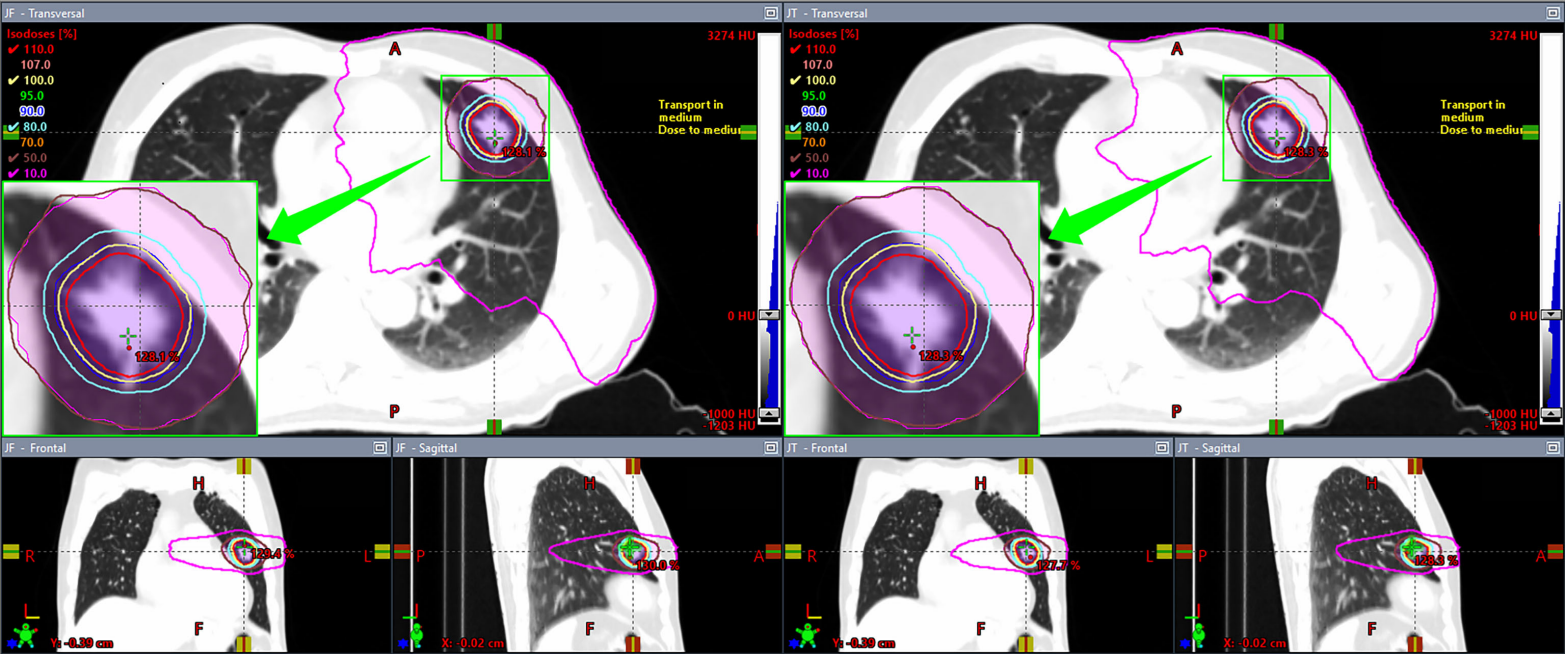
Comparison of dosimetric distributions between fixed-jaw (FJ; left panel) and jaw tracking (JT; right panel) plans of a patient.

All plans complied with the RTOG 0915 protocol (6, 7). The comparison between the dosimetric parameters of JT and FJ plans is summarized in **Table 1**. V_50%_, R_50%_, D_2cm_, and all parameters of the lung showed a statistically significant improvement in JT plans as opposed to FJ plans, while the number of MU in JT plans was higher by an average of 1.9%. No significant difference was found for V_100%_, CI, and the parameters of the spinal cord, esophagus, and trachea. The dosimetric distributions of a random patient with a PTV of 16.01 cc are shown in **Figure 1**. The purple-colored structure indicates the 50% prescription isodose volume of the JT plan, and the subfigure in the left panel shows the difference in the 50% prescription isodose volume between JT and FJ plans.

**Table 1:** Comparison between jaw tracking (JT) and fixed-jaw (FJ) plans. The data are shown in mean value ± standard deviation, along with the Wilcoxon signed-rank test *p*-values.

	Parameter	JT plan	FJ plan	*p*-value
PTV	V_100%_ (cc)	21.02 ± 15.28	21.03 ± 15.31	0.666
	V_50%_ (cc)	80.59 ± 50.56	82.19 ± 51.93	<0.001*
	CI	0.99 ± 0.02	0.99 ± 0.02	0.909
	R_50%_	4.00 ± 0.47	4.07 ± 0.47	<0.001*
	D_2cm_ (cGy)	2,018.22 ± 209.72	2,035.51 ± 213.48	0.006*
Lung	V_20Gy_ (%)	2.82 ± 1.67	2.85 ± 1.69	0.001*
	D_1,500cc_ (cGy)	34.77 ± 26.04	35.21 ± 26.55	<0.001*
	D_1,000cc_ (cGy)	88.42 ± 60.99	89.44 ± 61.62	0.001*
Spinal cord	D_0.35cc_ (cGy)	683.59 ± 296.19	671.16 ± 287.52	0.285
Esophagus	D_5cc_ (cGy)	357.7 ± 243.02	358.8 ± 242.57	0.472
Trachea	D_4cc_ (cGy)	817.41 ± 516.26	806.8 ± 498.88	0.177
Monitor unit	MU	3,701.98 ± 217.39	3,633.75 ± 236.70	0.001*

*Statistically significant (p < 0.05).

PTV, planning target volume.

**Figure 2:**
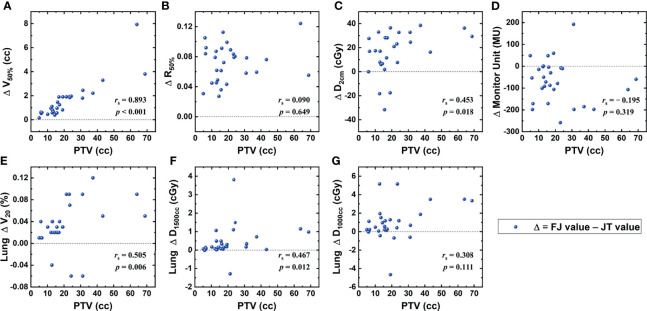
Correlations between the planning target volume (PTV) and the differences in dosimetric parameters between fixed-jaw (FJ) and jaw tracking (JT) plans, including V_50%_ of the PTV **(A)**, R_50%_ **(B)**, D_2cm_ **(C)**, monitor unit (MU) **(D)**, and V_20Gy_ **(E)**, D_1,500cc_ **(F)**, and D_1,000cc_ **(G)** of the lung.

**Figure 2** shows the correlations between the PTV and the differences in dosimetric parameters of FJ and JT plans that achieved statistical significance. As indicated in **Figure 1**, the PTV was strongly correlated with the difference in V_50%_ of the PTV, moderately correlated with that in V_20Gy_ of the lung, and weakly correlated with those in D_2cm_ and D_1,500cc_ of the lung.

**Figure 3:**
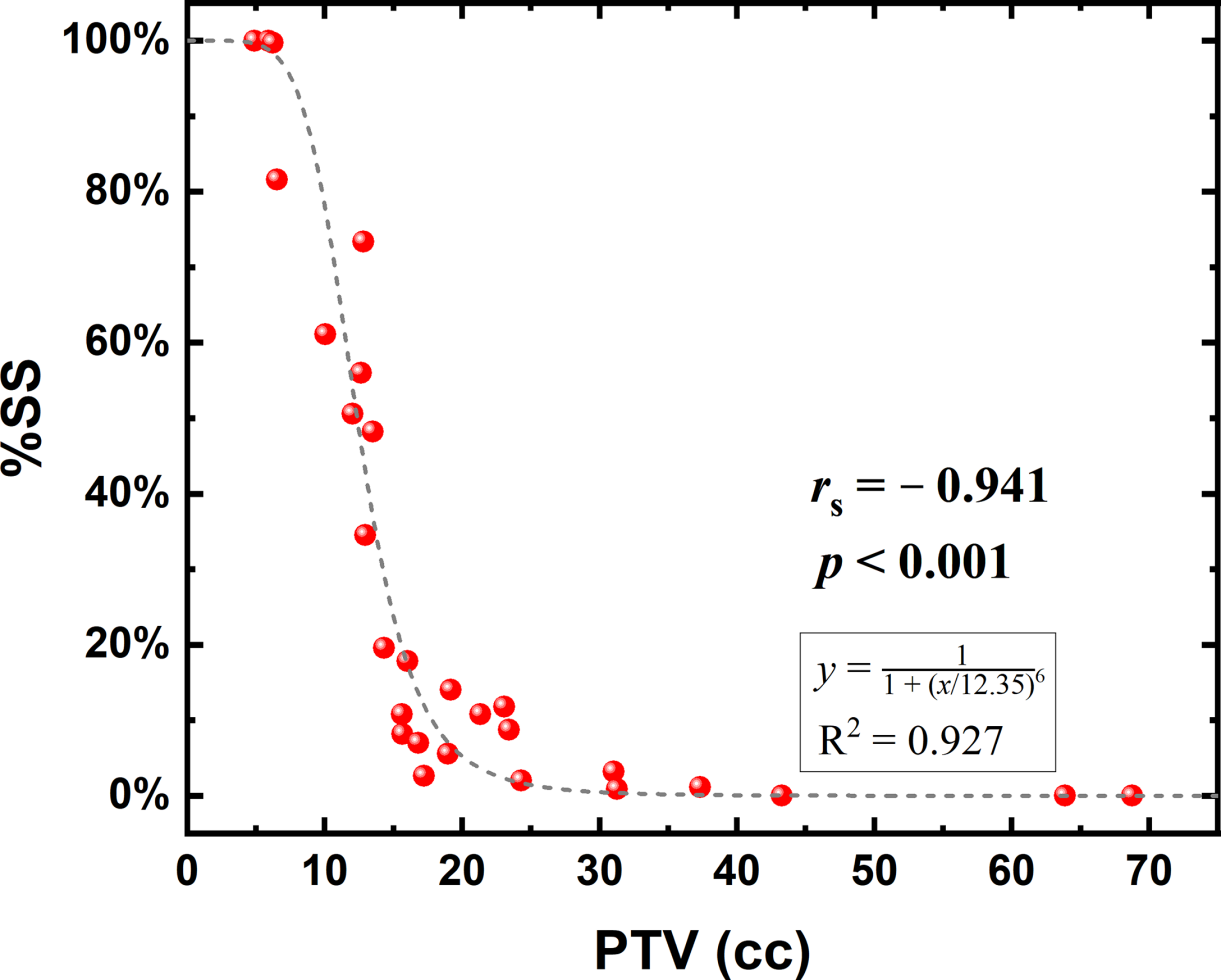
Correlation between the planning target volume (PTV) and the percentage of small subfields (%SS) in jaw tracking (JT) plans.

**Figure 3** shows the correlation between %SS and the PTV, including the corresponding curve fitting results, yielding a coefficient of determination (R^2^) of 0.927. The expected %SS values are basically 100% and 0% for PTVs below 6 cc and above 35 cc, respectively, and a near-linear correlation could be seen from 6 to 20 cc.

**Figure 4:**
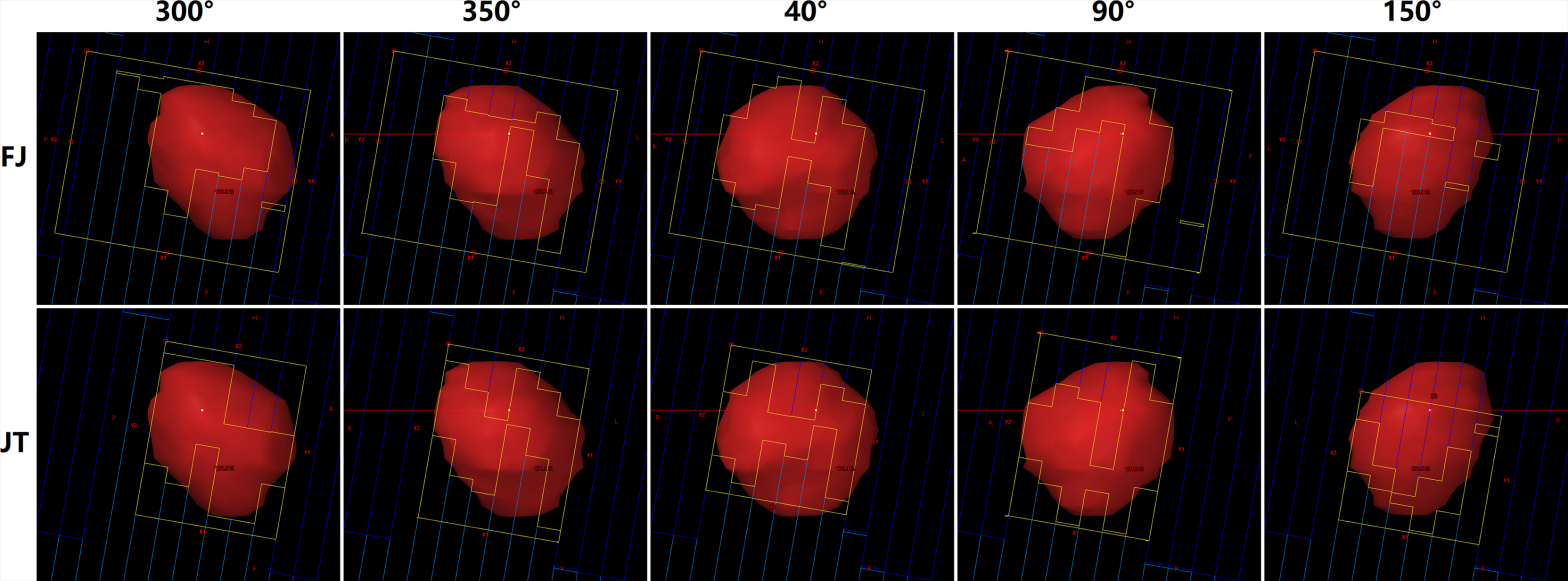
Beam’s eye views at five gantry angles in fixed-jaw (FJ; upper panel) and jaw tracking (JT) plans (lower panel) of a patient.

**Figure 4** displays the difference of jaw-defined subfield sizes at several gantry angles between JT and FJ plans of a random patient with a PTV of 16.01 cc. The average subfield size was reduced to approximately 45% with the use of JT technique for this patient.

## Discussion

This study investigated the differences in dosimetry and delivery accuracy between JT and FJ plans. One selection strategy by using PTV as a criterion was proposed to choose between JT and FJ techniques for lung SBRT.

A high-dose fall-off is vital to ensure an optimal normal tissue sparing in SBRT. Several studies have proposed that the JT technique could reduce the doses to OARs adjacent to the target with the reduction in the leakage and transmission of the MLC, potentially improving the dose fall-off away from the target (18, 22, 27). In the present study, **Table 1** and **Figure 1** show that the dosimetric improvement with the use of JT was in agreement with previous studies (19–22). Moreover, the PTV was found to be related to the magnitude of the improvements, which, to the authors’ knowledge, has not been investigated.

Compared with FJ plans, significant improvements were found for V_50%_, R_50%_, D_2cm_, and V_20Gy_, D_1,500cc_, and D_1,000cc_ of the lung in JT plans. These parameters were all associated with relatively lower dose levels, implying that the JT technique is primarily helpful for the protection of parallel organs where low-dose-volume parameters are more important, such as lung. As the JT technique is characterized by the reduction in leakage and intra-leaf transmission in the MLC, the low-dose region could be somewhat more directly affected, whereas the high-dose region mainly located at the center of the radiation field may be less sensitive. As indicated in **Table 1**, the V_100%_, CI, and near-maximum dose parameters of the spinal cord, esophagus, and trachea showed no significant differences between FJ and JT plans. In addition, the number of MU in JT plans were higher by an average of 1.9%, implying a small increase in the plan complexity. Among the parameters where significant improvements were found in JT plans, the magnitudes of the improvements in V_50%_, D_2cm_, and V_20Gy_ and D_1,500cc_ of the lung showed positive correlations with the PTV, as indicated in **Figure 1**. For smaller PTVs, the dosimetric differences between JT and FJ may be relatively indistinguishable, and the choice between JT and FJ should be primarily based upon other factors, such as delivery accuracy.

A high level of delivery accuracy is crucial for the implementation of SBRT. Due to the extensive use of small-field dose delivery in SBRT and the difficulties in measurements of small field, a number of reviews, guidelines, and recommendations have been published on the subject of small-field dosimetry, such as the IPEM report 103 (12), the IAEA-AAPM TRS-483 code of practice (13), and the more recent AAPM TG-155 report (14). However, due to limited resources or lack of qualified staff, many treatment centers may not be able to perform reliable small-field measurements, and the avoidance of uncertainties would be practical. For the Eclipse TPS, the small-field beam data will not affect the calculation results for the MLC-defined field where MLC is below jaws in the linac because the backscatter is determined from the jaw-defined field (15). When small jaw-defined fields are used, the measured output factor may affect the calculation accuracy for small-field cases (15). In JT plans with a small PTV, potential uncertainties could be brought by small subfield calculations (23–25). Therefore, it has been suggested that the FJ technique should be used in small-field planning to achieve minimal calculation uncertainties (26, 27).

In principle, to assess the difference in delivery accuracy between FJ and JT plans, measurement-based dose verification should be performed. Small-field uncertainty is one of the main contributors to the discrepancy between calculated and delivered doses. In accordance with the corresponding guidelines and recommendations (12–14, 28), the delivered dose for several FJ and JT plans was measured in the present study. However, due to the large uncertainties in the small-field data acquisition, TPS commissioning, and dose verification, obtaining reliable results to demonstrate the differences in the delivery accuracy between FJ and JT plans was difficult. Besides, discrepancies are inevitable in the TPS commissioning among linacs and treatment centers, and such could further undermine the validity of measurements. Therefore, the quantity %SS was used as a surrogate for the small-field uncertainty, and a higher %SS value indicates a potentially lower delivery reliability.

**Figure 3** shows that while using JT in VMAT planning, the variation of %SS occurred primarily in the PTV range of 6–20 cc, where a near-linear decrease was found with increasing PTV. As indicated above, the benefit of JT for small PTV is relatively trivial, and the delivery accuracy is a more important factor to be considered. Therefore, a threshold of PTV could be used as a decisive criterion for the use of JT. For example, the PTV should be at least approximately 12.5 cc for an expected %SS <50%, at least 15 cc for an expected %SS <20%, at least 18 cc for an expected %SS <10%, and at least 20 cc for an expected %SS <5%. With a PTV higher than 35 cc, the occurrence rate of small subfields in VMAT plans could be considered zero.

In addition, the intra-fraction motion of the target may cause a dose deviation between the calculated and delivered dose due to dose blurring and interplay effects between MLC and tumor motion (29–31), especially for a small-sized target (32, 33). Besides, the above results could also be affected by other factors, such as the plan complexity and tumor shapes (34–36). In the present study, an MU constraint and moderate ASC were used to achieve a relatively lower plan complexity and potentially reduce the %SS. Moreover, an irregular tumor with certain volume could lead to a higher %SS. Thus, this criterion should be applied on the basis of clinical circumstances. Although statistically significant dosimetric benefits were demonstrated in the above results, further clinical investigation is required to verify the outcome.

## Conclusions

In this study, the correlation between the magnitude of dosimetric improvement of implementing JT and the PTV in VMAT planning for lung SBRT was investigated, and a quantity %SS was proposed, i.e., the ratio of small subfields in VMAT plans, as a surrogate for the uncertainty in small-dose dosimetry. The PTV was found to be positively correlated with the dosimetric benefits of JT and negatively correlated with the %SS. By taking the benefits of JT and the small-dose uncertainty into account altogether, a selection strategy between JT and FJ was proposed using a PTV threshold.

## Data Availability Statement

The raw data supporting the conclusions of this article will be made available by the authors without undue reservation.

## Ethics Statement

Written informed consent was not obtained from the individual(s) for the publication of any potentially identifiable images or data included in this article.

## Author Contributions

Conception and design: HW. Acquisition of data: KC, YL, WX, CG, XY, and LW. Analysis and interpretation of data: WS, YS, and HW. Drafting of the article: WS and HW. Critical revision of the article: LD and HW. All authors contributed to the article and approved the submitted version.

## Funding

This work was supported by the Jilin Scientific and Technological Development Program (No. 20200601005JC) and the Jilin Province Special Project of Medical and Health Talents (No. JLSCZD2019-032).

## Conflict of Interest

The authors declare that the research was conducted in the absence of any commercial or financial relationships that could be construed as a potential conflict of interest.

## Publisher’s Note

All claims expressed in this article are solely those of the authors and do not necessarily represent those of their affiliated organizations, or those of the publisher, the editors and the reviewers. Any product that may be evaluated in this article, or claim that may be made by its manufacturer, is not guaranteed or endorsed by the publisher.
